# Short-chain fatty acid fermentation products of the gut microbiome: implications in autism spectrum disorders

**DOI:** 10.3402/mehd.v23i0.19260

**Published:** 2012-08-24

**Authors:** Derrick F. MacFabe

**Affiliations:** Director: The Kilee Patchell-Evans Autism Research Group, Departments of Psychology (Neuroscience) and Psychiatry, Division of Developmental Disabilities, Lawson Research Institute, University of Western Ontario, London, ON, Canada, N6A 5C2

**Keywords:** autism, mitochondria, Clostridia, Desulfovibrio, propionic acid, butyric acid, carnitine, neuroinflammation, oxidative stress, glutathione, gap junctions, microbiome, PUFA, epigenetics

## Abstract

Recent evidence suggests potential, but unproven, links between dietary, metabolic, infective, and gastrointestinal factors and the behavioral exacerbations and remissions of autism spectrum disorders (ASDs). Propionic acid (PPA) and its related short-chain fatty acids (SCFAs) are fermentation products of ASD-associated bacteria (*Clostridia*, *Bacteriodetes*, *Desulfovibrio*). SCFAs represent a group of compounds derived from the host microbiome that are plausibly linked to ASDs and can induce widespread effects on gut, brain, and behavior. Intraventricular administration of PPA and SCFAs in rats induces abnormal motor movements, repetitive interests, electrographic changes, cognitive deficits, perseveration, and impaired social interactions. The brain tissue of PPA-treated rats shows a number of ASD-linked neurochemical changes, including innate neuroinflammation, increased oxidative stress, glutathione depletion, and altered phospholipid/acylcarnitine profiles. These directly or indirectly contribute to acquired mitochondrial dysfunction via impairment in carnitine-dependent pathways, consistent with findings in patients with ASDs. Of note, common antibiotics may impair carnitine-dependent processes by altering gut flora favoring PPA-producing bacteria and by directly inhibiting carnitine transport across the gut. Human populations that are partial metabolizers of PPA are more common than previously thought. PPA has further bioactive effects on neurotransmitter systems, intracellular acidification/calcium release, fatty acid metabolism, gap junction gating, immune function, and alteration of gene expression that warrant further exploration. These findings are consistent with the symptoms and proposed underlying mechanisms of ASDs and support the use of PPA infusions in rats as a valid animal model of the condition. Collectively, this offers further support that gut-derived factors, such as dietary or enteric bacterially produced SCFAs, may be plausible environmental agents that can trigger ASDs or ASD-related behaviors and deserve further exploration in basic science, agriculture, and clinical medicine.

Autism spectrum disorders (ASDs) are a family of neurodevelopmental disorders of rapidly increasing incidence (1) that are characterized by impairments in communication and social interaction along with restrictive and repetitive behaviors.

The brain tissue of patients with autism shows subtle developmental abnormalities, specifically in those areas concerned with language, facial expression, movement, and social behavior (2). Individuals with autism may show enlarged brain size in the first few years of life, with altered migration of cortical, amygdalar, and cranial nerve motor neurons, as well as cerebellar neurons (3). Cell counts have shown that compared with controls, brain samples of patients with autism contain smaller neurons with increased cell density in cortical, limbic, and cerebellar regions (4), possibly because of altered neurogenesis (5), apoptosis (6), neural cytoarchitecture (7, 8), or a combination of these factors. There is a growing interest in examining ASDs as a disorder of glial cell function. Glial cells are of great importance in both the developing and mature nervous system, particularly with respect to cell–cell interactions during neural migration, and synaptic plasticity (9). Glia form a functional syncytium necessary for the maintenance of a stable neural microenvironment, especially during periods of increased metabolic stress (10). Glial abnormalities may manifest as an overall increase in white matter thickness (11), increased thickness of the external capsule and increased water content in the white matter (12). These observed abnormalities in brain morphology are accompanied by increased CNS immune activity, including increases in reactive astrocytes and activated microglia in brains of patients with ASD, as well as the elevation of proinflammatory cytokines in the cerebral spinal fluid. These findings have been demonstrated in both young and older patients, suggesting that an inflammatory process may be present throughout the life span of individuals with autism (13). Similar findings of heightened immune activity (i.e. increased Th2 cytokine levels) have also been demonstrated in peripheral blood monocytes from patients with ASDs (14).

To date, the majority of research has focused on genetic causes of ASD, mainly on developmental abnormalities in synaptic organization, neuromigration, and neurotransmission (15). However, the findings that genetic syndromes appear to account for only 6–15% of ASD cases, coupled with the observation of discordance of severity among monozygotic twins (16), indicate a solely genetic cause to be unlikely. An alternative approach is to examine ASDs as a whole body condition, with many comorbidities involving immune, metabolic, and gastrointestinal abnormalities, that may have a variable symptomatic course (17–22). Recent studies show widespread dysfunction in immune regulation, detoxification, environmental exposures, redox regulation/oxidative stress, and energy generation (19–22) that affect many organ systems, including the brain. Furthermore, the recent findings of genetic mutations, such as the MET receptor tyrosine kinase and protein kinase Cβ genes, involved in brain development, immune function, and ability to recover from gastrointestinal insults (23, 24), and the X-linked 6-*N*-trimethyllysine dioxygenase (TMLHE) gene, involved in carnitine/fatty acid metabolism (25), raise the strong possibility that genetic sensitivities to environmental factors may underlie the pathophysiology of the disorder in at least a subset of patients.

## Is autism an acquired disorder of mitochondrial dysfunction?

Disorders of mitochondrial function, and their heterogeneous expression in different tissues or within families, fulfill most of the criteria for widespread multiorgan dysfunction and sensitivity to many diseases (26, 27). Classic mitochondrial disease is overrepresented in patients with ASDs, encompassing 5% of the ASD population (28). More intriguingly, an extensive meta-analysis by Rossignol and Frye also found that about 30% of children in the general ASD population exhibit biomarkers consistent with mitochondrial disease (22), including a relative carnitine deficiency and altered lactate/pyruvate ratios. Furthermore, a recent study reported that 80% of the children with ASDs showed altered electron transport chain in lymphocytes compared with neurotypic controls (29). Mitochondria are central to this theme as polymorphisms in mitochondrial genes, which can be inherited or acquired from early prenatal environmental insults, can result in altered susceptibility to many diseases (26, 27). Similarly, abnormalities associated with ASDs such as glutathione deficiency (30), increased oxidative stress, elevated concentrations of TNF-α (31–33), and methylation abnormalities could further impair mitochondrial function. The fact that classic mitochondrial disorder is overrepresented but does not account for the high occurrence of mitochondrial biomarker alterations in this population may indicate that mitochondrial dysfunction in ASDs may be at least partly environmentally acquired. This is important in light of a rapidly growing incidence of ASDs (1) and the growing consensus that the systemic abnormalities seen in ASDs may arise from environmental triggers (34) in genetically sensitive subpopulations (35, 36).

Mitochondrial dysfunction can result from a broad assortment of environmental exposures that have been implicated in the development of ASDs. Possible agents, which are not mutually exclusive, include heavy metals (37–40), chemicals (41), polychlorinated biphenyls (42), pesticides (43–45), and finally enteric metabolic products of ASD-associated intestinal bacteria (46–53).

## The ‘inner’ outer environment, the enteric microbiome as a source of environmental triggers of ASDs

The human digestive tract is host to a complex array of intestinal bacterial florae, coined the microbiome, which outnumber host cells at least 10 to 1. The microbiome produces an array of bioactive metabolic products capable of entering systemic circulation. It is important to note that the enteric microbiome and its metabolic products are not static and can be altered throughout the life cycle of the individual, particularly throughout the first 18 months of life (54). The metabolic products from the gut microbiome can have profound and dynamic effects on host metabolism, immune function, and gene expression in many organ systems, including the CNS (46, 55–60). In addition, it is also important to consider the recent impact of the alteration of the human microbiome and its metabolites through the introduction of the high calorie Western diet, coupled with the high exposure of antibiotics and disinfectants to human beings, animals, and plants, as a possible source of environmental triggers of many diseases of increasing incidence (59), including ASDs. This is particularly evident from human populations migrating to Western societies, such as the Somali diaspora, who appear to have a much higher incidence of ASDs than in their country of origin (61).

Given the high number of reports of antibiotic exposure, hospitalization, and gastrointestinal disturbances (62–64) in many patients with ASDs, our laboratory has been examining the neurobiological effects of microbiota-produced short-chain fatty acids (SCFAs), such as propionic acid (PPA) (46–53). PPA is a fermentation product of many bacteria, including some enteric species (i.e*. Clostridia*, *Desulfovibrio*, and *Bacteroidetes*) overexpressed in stool samples from patients with ASDs (65, 66). PPA levels are also elevated in the stool samples of patients with ASDs (67). This has led us to propose PPA as a possible environmental trigger of ASDs. We have found that when administered to rodents, brief intracerebroventricular (ICV) infusions of PPA and its related enteric SCFAs produce many behavioral, electrographic, neuropathological, and biochemical changes, consistent with an animal model of ASDs.

## Neurobiological effects of enteric SCFAs

PPA is found in the gut, along with other SCFAs, such as acetate and butyrate, each of which are major metabolic products of enteric bacteria, following fermentation of dietary carbohydrates and some amino acids (58, 68, 69). PPA is also produced by *Propionibacteria* present on the skin, known to cause acne (70), and many bacteria present in the oral mucosa, responsible for gingival inflammation (71, 72). PPA is well known in animal husbandry, where it is the main metabolic product in ruminant cellulose digestion (73), may be a sex pheromone (74), and is involved in lactation (75). It is also naturally present in a variety of foodstuffs (i.e. cheese) (73) and is commonly used as a preservative (antifungal) in many processed foods, particularly in refined wheat and dairy products (76). However, the majority of PPA is produced in the gut lumen by intestinal bacteria. PPA, being a weak organic acid, exists in ionized and non-ionized forms at physiological pH, thus allowing it to readily cross the gut–blood barrier, and is principally metabolized in liver. It also crosses the blood–brain barrier and enters the CNS (77). In addition, PPA gains access to the CNS via monocarboxylate transporter uptake in the gut lumen and cerebrovascular endothelium, which actively transport many carboxylic acids, particularly PPA and ketones. PPA is also a specific ligand of many G-coupled SCFA receptors (GPR41, 43) (78–80). PPA and other SCFAs are taken up by glia and, to a lesser extent, neurons once they enter the CNS (81, 82), where they are thought to comprise a major energy source in cellular metabolism, particularly during early brain development (81–83). PPA and other SCFAs (i.e. butyrate) affect diverse physiological processes such as cell signaling (84), neurotransmitter synthesis and release (85), free radical production, mitochondrial function, (86) lipid metabolism (87), immune function (88), gap junction gating, intracellular pH maintenance (89), and modulation of gene expression through phosphorylation and histone acetylation (90).

Although PPA may be beneficial at appropriate levels, such as improving insulin sensitivity, lowering cholesterol, and reducing food intake (69), excessive PPA may have many negative effects on health and behavior. For example, a number of inherited and acquired conditions, such as propionic/methylmalonic acidemia, biotinidase/holocarboxylase deficiency, ethanol/valproate exposure, and, as discussed, mitochondrial disorders, are all known to result from elevations of PPA and other SCFAs, partly through the formation of propionyl coenzyme A (CoA) and sequestration of carnitine (20, 46, 51, 91). Collectively, these conditions present at varying ages with developmental delay and regression, seizure/movement disorder, metabolic acidosis, and gastrointestinal symptoms, which also change in severity with fluctuating PPA levels and markers of mitochondrial dysfunction that are somewhat reminiscent of ASDs (86, 92, 93).

Propionic acidemia is caused by deficient activity of either one of two non-identical subunits of the biotin-dependent enzyme propionyl CoA carboxylase (94). This mitochondrial enzyme is responsible for the breakdown of PPA and other SCFAs, as well as a number of amino acids. The disorder may be 10 times more common than previously reported, and there are multiple mutations and variable metabolizers in many populations (95, 96).

Patients with propionic acidemia clinically present with life-threatening illness during the neonatal period, characterized by vomiting, severe metabolic acidosis, and hyperammonemia. Neurological symptoms include developmental delay, seizure, choreathetoid movements, and dystonia. Interestingly, some other patients, including identical twins of more severely affected siblings, may present later in life with varying severities of the disorder, often without measurable increases in blood or urine PPA or metabolites. Treatment includes reduction of carbohydrate and protein contents in the diet, eradication of PPA-producing bacteria, and carnitine supplementation to improve PPA clearance, which have some benefit (46, 86, 92, 93).

Thus, PPA and its related SCFAs have broad effects on cellular systems, providing a potential mechanism where metabolic end products of the enteric microbiome can alter host physiology and behavior (46, 60). This and other evidence led us to propose that increased PPA exposure at key neurodevelopmental periods is a major environmental trigger of the brain and behavioral changes observed in ASDs.

## How are dietary and gastrointestinal factors related to autism? – microbiome production of enteric fatty acids as environmental triggers

There is a growing interest suggesting a link between dietary and/or gastrointestinal factors and the worsening and, in some cases, improvement of ASD symptoms, but the mechanisms responsible for this remain elusive. As indicated, SCFAs, such as PPA, are produced by many gut bacteria by the breakdown of dietary carbohydrates and amino acids, particularly from wheat products (97). Of particular relevance are the *Clostridia* and *Desulfovibrio*, which have been proposed as infectious causes of ASDs (64, 66). Clostridial species, a family of heterogeneous anaerobic, spore-forming, gram-positive rods (98), are major gut colonizers in early life and are producers of PPA.

*Clostridia* as spore formers are particularly resistant to most antibiotics used for routine perinatal and early childhood infections, and some species are a cause of major hospital- and, recently, community-acquired communicable disease (i.e. *C. difficile*-induced colitis) (98). Interestingly, spore-forming anaerobes and microerophilic bacteria, particularly from clostridial species, have been shown to be elevated in patients with ASDs (66, 99, 100). Recently, Finegold isolated distinct species of *Desulfovibrio*, a gram-negative, aerotolerant, non-spore former, from the stool of patients with ASDs, and, to a lesser extent, non-affected siblings. Interestingly, in addition to producing PPA following fermentation of peptones, *Desulfovibrio* is resistant to most common antibiotics and produces the gasotransmitter and potential mitochondrial toxin, hydrogen sulfide (101–103). He has suggested eradication of these organisms with oritavancin and aztreonam as a possible treatment of ASDs (64). Furthermore, ASDs may show comorbidity with a variety of gastrointestinal disorders, such as alterations in gut motility, intestinal lesions and increased intestinal permeability, bacterial dysbiosis, impaired carbohydrate digestion/absorption, reflux esophagitis, and ileal hyperplasia (46, 104, 105). An association between long-term antibiotic use, hospitalization, abdominal discomfort and the onset of ASD symptoms after normal or near-normal development has also been reported (62, 63, 100, 106). These findings raise the possibility that gut-born factors secondary to alteration of the gut microbiome by antibiotics or diet may influence brain function and symptomology in patients with ASDs. Moreover, a compromised gut–blood barrier (i.e. an acquired colitis) or impaired colonocyte energy metabolism (102), which use SCFAs as an energy substrate and act as a metabolic ‘sink’, may allow for greater systemic and CNS access for such compounds (59, 107).

PPA is also known to have a number of direct effects on gastrointestinal physiology. As reviewed in the study by MacFabe et al. (46), PPA increases contraction of colonic smooth muscle, dilates colonic arteries, activates mast cells, increases the release of serotonin from gut enterochromaffin cells, and reduces gastric motility and increases the frequency of contractions, reminiscent of the clinical observations of gut dysmotility in patients with ASD. PPA levels increase in infant colonic contents postnatally and following formula feeding opposed to breast feeding (54). Interestingly, intracolonic infusions of PPA, mimicking bacterial overgrowth, can induce an experimental colitis in infant rats but not later in life (108). However, ascertaining PPA production *in vivo* is difficult, as secondary to both passive and active diffusion of SCFAs from the gut lumen, and intracellular concentration levels in stool are often a poor indicator of SCFA production by gut bacteria and may change rapidly with diet (58).

Taken together, PPA appears to circumstantially possess the necessary properties to interfere with gastrointestinal activity in a manner similar to the abnormalities observed in ASDs. In support of this, reports from parents of children with ASDs suggest that behavioral and gastrointestinal symptoms increase when their children ingest refined food products that provide high carbohydrates for bacterial fermentation to produce PPA or also contain PPA as a preservative. Furthermore, behaviors and gut symptoms improve following the elimination of these products from the diet (17, 106) or the eradication of PPA-producing bacteria by broad-spectrum antibiotics (109). Moreover, symptom exacerbation associated with propionic acidemia and its related conditions bears some resemblance to that reported in ASDs (86, 92, 93). Stool (67) and serum studies from patients with ASDs provide further evidence linking PPA to the condition, as patients with ASDs have metabolic dysfunction, including impairments in B12, glutathione, or carnitine metabolism (110, 111) and mitochondrial disorder/dysfunction (20), which are consistent with the effects of PPA on cellular metabolism (46, 51, 53).

## The PPA rat model of ASDs – behavioral and electrographic findings

Animal models allow for the examination of factors involved in human disorders, such as ASDs, using experiments that cannot be conducted in humans. The development of such models is essential and permits the experimental examination of the effects of suspected environmental agents on the pathophysiology and core symptoms and comorbidities of ASDs. There have been a number of valid animal models of ASDs, concentrating on genetic knockouts of key neurodevelopmental processes (112, 113) and also on exposures to environmental factors such as metals, drugs (valproic acid, terbutaline) (114, 115), viruses (116) and inflammatory agents (i.e. lipopolysaccharide, interleukin-6) (117). Each of these models causes some, but not all, behavioral or pathological changes reminiscent of ASDs. Therefore, to investigate the hypothesis that that elevated levels of PPA can induce bouts of behavioral, electrophysiological, neuropathological, and biochemical effects similar to those observed in ASDs, our initial research was concentrated on exposure of adult rats to brief pulsed ICV infusions of PPA and its related enteric SCFAs (i.e. acetate, butyrate), through chronic indwelling brain cannula. CNS exposures were initially done to test for a central, and not peripheral, action of these compounds, as it has been argued that the behaviors, dietary/gut symptoms, and metabolic findings in ASDs are solely due to responses to gastrointestinal pain, poor diet, or obsessional eating behavior and not a direct effect of enteric compounds on the function of CNS.

Impairments in social behavior, including abnormal play behaviors and other forms of social contact, are among the most prominent symptoms of ASDs (49, 118). Likewise, individuals with ASDs commonly suffer from various forms and degrees of cognitive impairment, including learning disabilities, restricted interests favoring objects vs. social interactions, and ‘rigid’ perseverative behavior, and insistence on ‘sameness’, and rituals (18, 119). Patients with ASDs often experience motor-related symptoms, such as hyperactivity, gait disturbances, and stereotyped movements, possibly grouping ASDs with the movement disorders (120, 121). There is increased incidence of seizure disorder in ASDs and other conditions associated with elevated levels of SCFAs (92, 122, 123). We thus tested the validity of the PPA model based on the above behavioral and electrographic criteria.

**Fig. 1:**
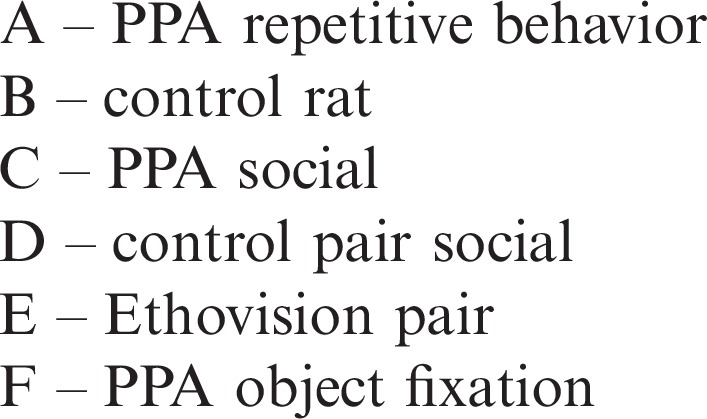
Behavioral videos of propionic acid infusions in rats (click headings to view videos). Single intracerebroventricular (ICV) infusions (4 µl of 0.26 M solution over 4 min) of propionic acid (PPA), a metabolic end product of autism-associated enteric bacteria, produce bouts of reversible hyperactive and repetitive behavior (A) in adult rats, compared with phosphate-buffered saline (PBS) vehicle infused control rat (B). Rat pairs infused with PPA show markedly reduced social interaction and play behavior (C), compared with pairs of rats infused with PBS vehicle (D), which show typical social behavior. Ethovision behavioral tracking of control and PPA-treated rat pairs (E), showing further evidence of PPA-induced hyperactive, repetitive and antisocial behavior. PPA-treated rat displays fixation on objects (F) and a specific object preferences (i.e. block vs. sphere). PPA-infused rats also show turning, tics, dystonia, and retropulsion and electrographic evidence of complex partial seizures and basal ganglial spiking, consistent with findings in patients with autism spectrum disorders.

Pulsed ICV infusions of PPA (4 µl of 0.052–0.26 M solution at pH 7.5 over a 1 min period) or control compounds (i.e. isomolar, acetate, butryate and propanol - the non-acidic alcohol analogue of PPA) were performed over a number of time courses (once weekly×5 weeks, once a day×5 day, twice daily for 7–14 days) into the cerebrospinal fluid of adult rats via chronic brain indwelling canullae (Fig. 1).

**Fig. 2:**
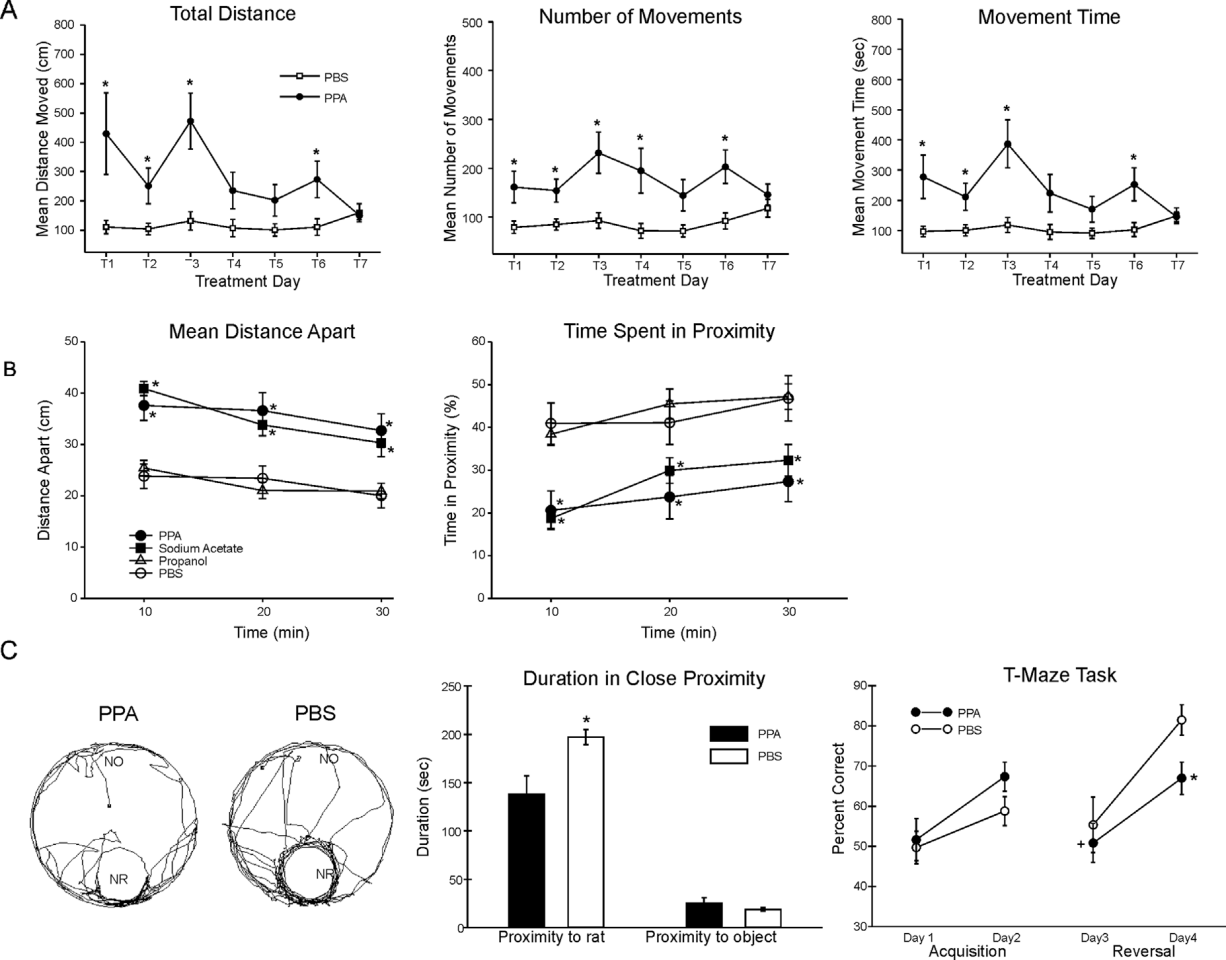
(A) Intracebroventricular (ICV) infusions of propionic acid (PPA) in adult rats increase repetitive locomotor activity (Versamax automated behavioral assay). Group mean values (±SEM) total distance, number of movements, and movement time in rats given 4 µl ICV infusions of phosphate-buffered saline (PBS) vehicle or PPA (0.26 M) twice daily for 7 days. BL = baseline session with no infusion; T1–T7 consecutive treatment days. *p<0.05. (B) Single ICV infusions (4 µl of 0.26 M solution over min) of PPA and isomolar acetic acid (SA) in adult rat pairs reduce social behavior (Ethovision automated behavioral assay). Behavior is measured as mean distance apart (cm) and time spent (%) in 5 cm proximity during dark hours. Data points represent group means of data collected during 10 min periods. Animals were placed in a large open field in same-drug pairs. Both PPA and SA pairs displayed a significantly greater mean distance apart and spent significantly less time in close proximity to each other, consistent with impaired social behavior found in autism, compared with phosphate-buffered saline (PBS) vehicle controls. Propanol the non-acidic alcohol analogue of PPA was without effect. *=different from PBS control group at *p*<0.05 or better. (C) Tracks representing object and socially related behavioral movement of adolescent rats receiving ICV infusions of PPA (4 µl of 0.26 M solution over 4 min) or PBS. More locomotion near the caged novel rat (NR) occurred in PBS rats than by PPA rats. Graphic representation of duration of time (s) spent in close proximity (within 18 cm) of the novel rat or novel object (NO). PPA rats showed less approach behavior and remain close to the novel rat less than PBS rats, indicative of object preference over social behavior, consistent with findings in patients with autism. Graphic representation of group mean (±SEM) percent correct turns in the T-maze task during acquisition (Days 1 and 2) and reversal (Days 3 and 4). + = significant difference from Day 2 performance; * = significant different from PBS control group. Figures modified with permission from MacFabe et al. (2008) and (2011), and Shultz et al. (2008). Figure 2A reproduced with permission from Science (American Association for the Advancement of Science). Figures 2B and 2C reproduced with permission from Elsevier Ltd.

PPA-infused rats showed bouts of increased repetitive locomotor activity, turning, retropulsion, tics, social impairment, perseveration, and restrictive preference for objects versus novel rats (46–53) (Fig. 2). These behaviors were rapidly induced within 1–2 min after single infusions, but were transient, lasting approximately 20–30 min, consistent with levels in bloods of propionic acidemia patients, and the half-life of PPA (124).

Interestingly, it was found that during the acquisition session in the Morris water maze and T maze, tests for visuospatial learning, the PPA-treated rats displayed mild or no impairment in maze acquisition but marked impairments following maze reversal (49, 50). In the case of the Morris water maze, when the location of the target platform was moved for the reversal session, the PPA-treated rats displayed marked cognitive impairments, often swimming toward the former location of the platform, consistent with ‘rigid’ perseverative behavior, which could be considered a deficit in ‘un-learning’.

**Fig. 3:**
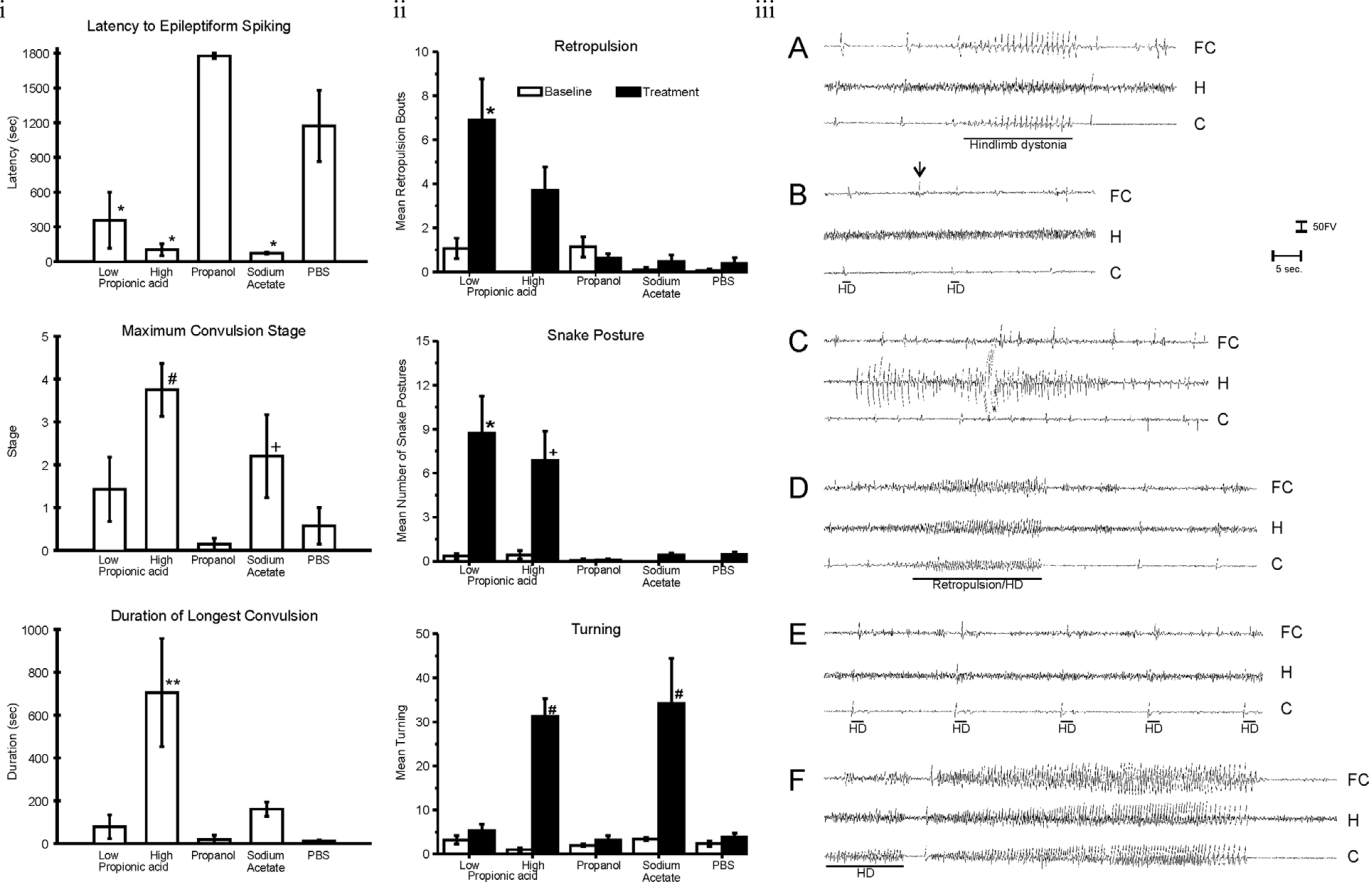
(I) Intracerebroventricular (ICV) propionic acid (PPA) infusions in rats induce electrographic and behavioral elicitation of kindled seizures and movement disorder. Kindled seizure manifestation in response to repeated weekly 4 µl ICV infusions of low (0.052 M) or high (0.26 M) PPA, isomolar acetic acid (SA), propanol, or phosphate-buffered saline vehicle (PBS) in adult rats: group mean (±SEM) data summed across the five initial testing sessions. Latency to epileptiform spiking was measured from the end of the ICV infusion to the start of seizure. Maximum convulsion stage was rated using the Racine kindling scale. Duration of the longest convulsion was of the longest continuous convulsion during a session. PPA and, to a lesser extent, SA induce a kindling response, while propanol, the non-acidic analogue of PPA, was without effect. * = different from propanol and PBS controls; # = different from low PPA, propanol control, and PBS control; + = different from propanol control; ** = different from all other groups. (II) Abnormal behaviors in response to ICV infusions: group mean frequency (±SEM) of behaviors per baseline or initial test session. Either one or both doses of PPA increased the abnormal behaviors relative to control treatments. Only PPA produced dystonic (snake like), retropulsive behaviors. With the exception of sodium acetate, which increased turning, no control treatments increased abnormal behavior. Black bars indicate treated animals; white bars indicate PBS (vehicle)-treated animals. * = *p* < 0.05 or better vs. all control groups; # = *p* < 0.05 or better vs. low PPA, propanol, and PBS control. (III) Representative electrographic seizure records from rat in the high PPA group. (A) Session 2, short bout of epileptiform spiking accompanied by contralateral hindlimb dystonia coincident with spiking (event marker). Note spiking in frontal cortex and caudate but not dorsal hippocampus. (B) Session 3, single epileptiform spikes in caudate and frontal cortex but not hippocampus. Only the caudate spikes were accompanied by brief contralateral hindlimb dystonia (event markers), which led the frontal cortex spikes by approximately 500 ms. A prominent frontal cortex spike (arrow) is not accompanied by a spike in the caudate or by limb dystonia. (C) Session 5, bout of spiking in dorsal hippocampus not accompanied by corresponding bouts of spiking in frontal cortex or caudate or by limb dystonia. (D) Session 5, single epileptiform spikes occur first in the caudate and lead spikes in other traces. These are followed by a short bout of epileptiform spiking that is accompanied by brief retropulsion, followed immediately by contralateral hindlimb dystonia, which ends coincident with the end of the bout of spiking (event marker). (E) Session 5 at approximately 1 min after the records in D, single epileptiform spikes in all three traces, with the caudate spikes leading the spikes in the other traces. Each spike is accompanied by brief contralateral hindlimb adduction and immediate dystonia (event markers). (F) Session 5 at approximately 6 min after the records in E, short bout of epileptiform spiking with hindlimb dystonia beginning coincident with caudate spiking (event marker), with frontal cortex and hippocampal spiking beginning after onset of caudate spiking and hindlimb dystonia. This is followed 2 s later by the beginning of the first sustained kindled seizure displayed by this rat (duration = 35s), which was accompanied by the first conventional kindled convulsion (Stage 2), which did not include limb dystonia. C = caudate; FC = frontal cortex; H = dorsal hippocampus; HD = hindlimb dystonia. Amplitude calibration = 50 µv; time marker = 5 s. Figures modified with permission from MacFabe et al. (2007). Reproduced with permission from Elsevier Ltd.

Using rats with stereotactically implanted indwelling cortical, hippocampal, and subcortical brain electrodes, repeated PPA exposures (once weekly×5 weeks) produced a kindling response in hippocampal/neocortical leads and complex-partial-seizure-like behavior, while tics, retropulsion, and dystonia were accompanied by sharp spiking in the basal ganglia. Infusions of butyrate and acetate produced similar, but less pronounced effects, while infusions of propanol were without effect (46) (Fig. 3). Current studies have found similar behaviors at PPA doses 1/20th of initial investigations (unpublished observations).

Thus, ICV infusions of PPA and, to a lesser extent, other SCFAs rapidly produce a number of striking behaviors that resemble those seen in patients with ASDs. In addition, many of these behaviors appear to reverse, consistent with the metabolic breakdown of PPA.

## Potential underlying mechanisms of PPA and their relation to autism – neurotransmitters

PPA and its related SCFAs are capable of gaining access to the brain and inducing widespread effects on CNS function (77), including neurotransmitter synthesis and release, calcium influx, intracellular pH maintenance, lipid metabolism, mitochondrial function, gap-junction-dependent intercellular gating, immune activation, and gene expression, which we have proposed may contribute to the behaviors and biochemical findings observed in the PPA animal model and ASDs (46).

Of interest, PPA is capable of altering dopamine, serotonin, and glutamate systems in a manner similar to that observed in ASDs (125–130), partly via potentiating intracellular calcium release (77, 131, 132). Furthermore, and of importance to the symptoms observed in both ASDs and the PPA model, similar alterations to the serotonin and dopamine systems have been implicated in abnormal social and motor behaviors (127–129). SCFAs, including PPA, may increase synthesis of dopamine and its related catecholamines through induction of tyrosine hydroxylase, a key enzyme in the synthesis of catecholamines (133). This may also occur peripherally in the adrenals and sympathetic ganglia, where they may contribute to the enhanced anxiety-like behavior and increased sympathetic tone and cardiovascular instability found in ASDs (134).

PPA and other SCFAs are also known to potentiate glutamatergic transmission, inhibit GABAergic transmission and increase the production of enkephalin (46, 130, 135), supportive of a potential mechanism for the enhanced excitation/reduced inhibition theory of ASDs (136). PPA is also known to modulate neurofilament phosphorylation/dephosporylation, important in neurodevelopment and neuroplasticity (137, 138).

## PPA-induced intracellular acidification, increased oxidative stress, impaired antioxidant capacity, and gap junction closure

The findings that PPA and its related fatty acids often induce similar behavioral effects, whereas isomolar infusions of propanol do not (46, 49, 50), make it tempting to speculate that the carboxylate functional group and/or acidic properties of PPA may be involved in its effects on behavior. Once low-molecular-weight organic acids are protonated in acidic conditions, they become more lipid soluble and, thus, gain access to the CNS, either by passive diffusion or active transport (77, 78, 80). Once they enter the CNS, they may concentrate within cells and induce intracellular acidification. Previous studies have found that PPA- and acetate-related acidosis in rats can alter social behavior (139). Furthermore, clinical metabolic acidosis from varied etiologies in humans often involves bouts of confusion and movement disorder similar in some respects to those found in ASDs (140). The effects of varying intracellular pH on cellular physiology are broad. Notably, PPA-induced intracellular acidification can inhibit mitochondrial function, which is vital for normal metabolism of other fatty acids (141). Intracellular accumulation of organic acids creates a buildup of acyl-coenzyme, which interrupts metabolism (141). PPA sequesters carnitine function and inhibits CoA function and, thus, impairs mitochondrial metabolism and energy production, leading to further decrease in cytoplasmic pH via the accumulation of other organic acids (131, 141). This is consistent with the impaired mitochondrial metabolism in ASDs, including carnitine deficiency, mitochondrial dysfunction, and systemic elevations of nitric oxide metabolites (20, 46, 142). In addition, valproate, an antiepileptic drug, structurally related to PPA, and a known prenatal risk factor for ASDs (143), similarly alters mitochondrial metabolism and causes the depletion of carnitine stores and encephalopathy (144, 145). Nitropropionic acid, another PPA derivative, is a potent inhibitor of mitochondrial function and produces a model of Huntington chorea when administered to rodents (146). The basal ganglia may be particularly sensitive to PPA and its derivatives, because of the region's high metabolic demand and damage in many movement disorders, including organic acidemias and mitochondrial disorders, thus offering an explanation of the tics, dystonias, and repetitive behaviors in these conditions and the PPA rodent model (46, 86).

**Fig. 4:**
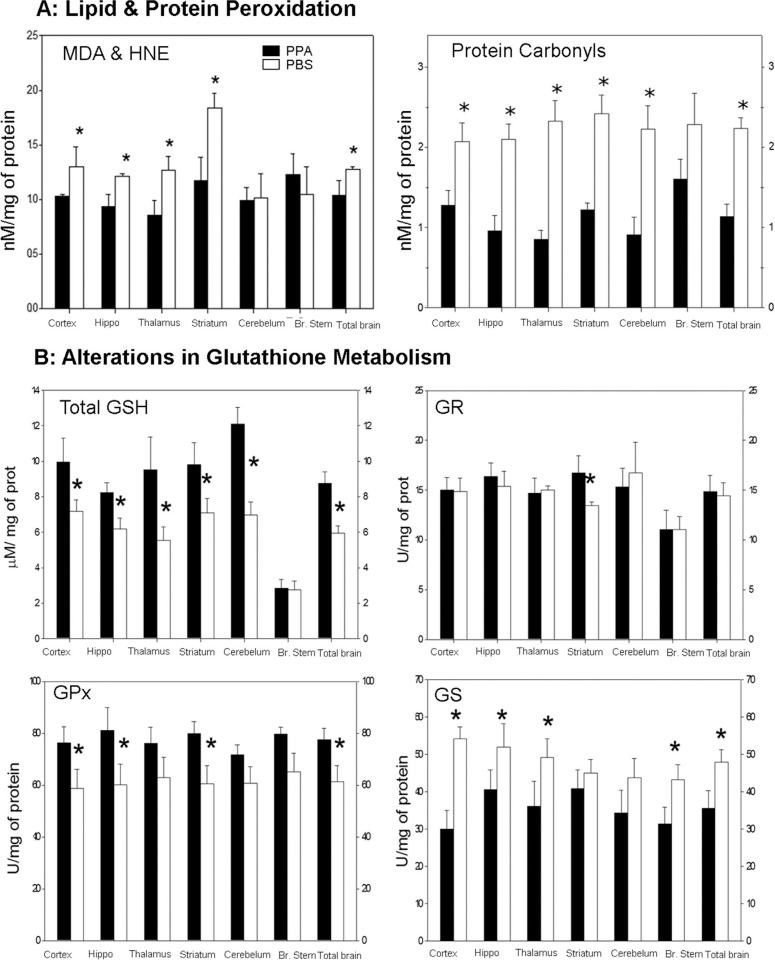
(A) Propionic acid (PPA) infusions in rats induces a significant increase in lipid and protein oxidation, supportive of increased oxidative stress via increased oxidant production and decreased antioxidant defenses in homogenates of discrete regions of rodent brain consistent with findings in patients with autism. Interestingly, brain stem appears relatively unaffected. (B) PPA-treated rats showed significantly decreased total GSH, a decreased activity of GPx, but an increase in the activity of GST. Conversely, the activity of GR was unchanged. These findings suggest that GSH may be involved in the metabolic clearance of PPA, or alternatively, the synthesis of GSH was impaired by PPA. The observed reduction of the activity of GPx may be due to the decreased availability of GSH, a cofactor of GPx or may reflect an overall reduction in antioxidant defenses induced by PPA. Black bars indicate PPA-treated animals; white bars indicate PBS (vehicle)-treated animals. Figures modified with permission from MacFabe et al. (2008). Reproduced with permission from Science (American Association for the Advancement of Science).

An impaired mitochondrial metabolic process similar to the one described could lead to a range of negative effects on the CNS. Of relevance, similar encephalopathic processes associated with increased oxidative stress, such as those found in organic acidemias, are known to produce symptoms consistent with findings in the PPA model and ASDs (86, 93, 147). Consistent with this hypothesis, biochemical analyses of homogenates of the brain sample from PPA-treated rats (4 µl of 0.26 M solution BID×7 days, ICV) demonstrated an increase in oxidative stress markers (protein carbonylation and lipoperoxidation), as well as abnormalities in glutathione-associated pathways, particularly in brain regions implicated in ASDs (46, 47, 148) (Fig. 4).

Glutathione participates in both antioxidant defense and xenobiotic detoxification over a broad range of environmental organic compounds and metals (149, 150), including those implicated in ASDs. Impairments in glutathione-associated pathways suggest reduced cellular defense and are considered markers of increased oxidative stress (149). Of particular interest is the evidence of genetic and acquired impairments in glutathione-associated pathways in patients with ASDs (150–152), suggesting a plausible mechanism for altered sensitivity to a wide range of environmental agents (metals, pesticides, drugs) proposed in ASDs (46, 47). Of note, acetaminophen, when given for common pediatric illnesses, may overwhelm glutathione metabolism and has been proposed as a possible trigger for ASDs (153–155). Furthermore, even brief exposures to agents that alter redox levels in cells early in development may change cellular developmental trajectory and ultimate cell fate, which may provide a plausible mechanism for neurodevelopmental alterations in ASDs (156). Overall, PPA-induced metabolic abnormalities and oxidative stress are consistent with findings from ASDs and ASD-related disorders.

Further to its capability to decrease intracellular pH, both directly via intracellular concentration and indirectly via inhibition of mitochondrial functions, we have proposed that a major effect of PPA is through the closure of gap junctions via intracellular acidification (46, 50, 157). As reviewed in MacFabe et al. (46), gap junctions are intercellular channels composed of connexin proteins that are gated by a number of factors influenced upon by PPA, including dopamine, calcium, and cytokines. Gap junctions play a major role in cellular differentiation, and in particular, peripheral nerve, cardiac, uterine, and gastrointestinal function. However, in the CNS, gap junction coupling is vital for the synchronization of neural electrical activity within discrete functional cell groups and is more extensive during early brain development and neuronal migration. Astrocytes are electrotonically connected by gap junctions, forming a syncytium to spatially buffer calcium, glutamate, and potassium (158), and apoptotic factors are capable of passing through these glial gap junctions (159, 160). Thus, closed glial gap junctions may render neurons hyperexcitable to rising extracellular potassium and glutamate (160), while closed neuronal gap junctions would be neuroprotective (159). In turn, this decrease in gap junction coupling may lead to inhibited cortical pruning in development, consistent with the larger brain size found in ASDs (46). Gap junction communication is involved in neurotransmission in areas that are implicated in seizure and movement disorders, such as the basal ganglia, prefrontal cortex, nucleus accumbens, and hippocampus. Intrastriatal injections of gap junction blockers produce stereotypical movements, hyperlocomotion, and disruption of motor sequencing in rodents (127, 161). Furthermore, gap junction knockout mice show abnormal brain development, exaggerated responses to neurotoxic insults, seizure disorder, and abnormal behaviors (162). Interestingly, gap junction blockers also inhibit tight junctions in many cellular systems (163), thereby possibly contributing to altered barrier function in vascular endothelium and gut in ASDs (107). Given these findings, it seems possible that PPA-induced alterations to gap junction function may contribute to electrophysiologic and motor impairments observed in both the PPA model and ASDs, but also in neural development, as well as systemic effects (i.e. gastrointestinal motility). These potential mechanisms are the subject of further study in our group.

However it is unclear at this stage whether these effects on pH and oxidative stress are causal to behavioral induction or the results of PPA dependent mechanisms such as neuroinflammation or altered lipid metabolism. Quite possibly they are not necessarily exclusive and may be mutually reinforcing, leading to a vicious cycle of these processes.

## Neuropathological effects of PPA infusions on inflammation/CREB induction/epigenetics/lipid transport

**Fig. 5:**
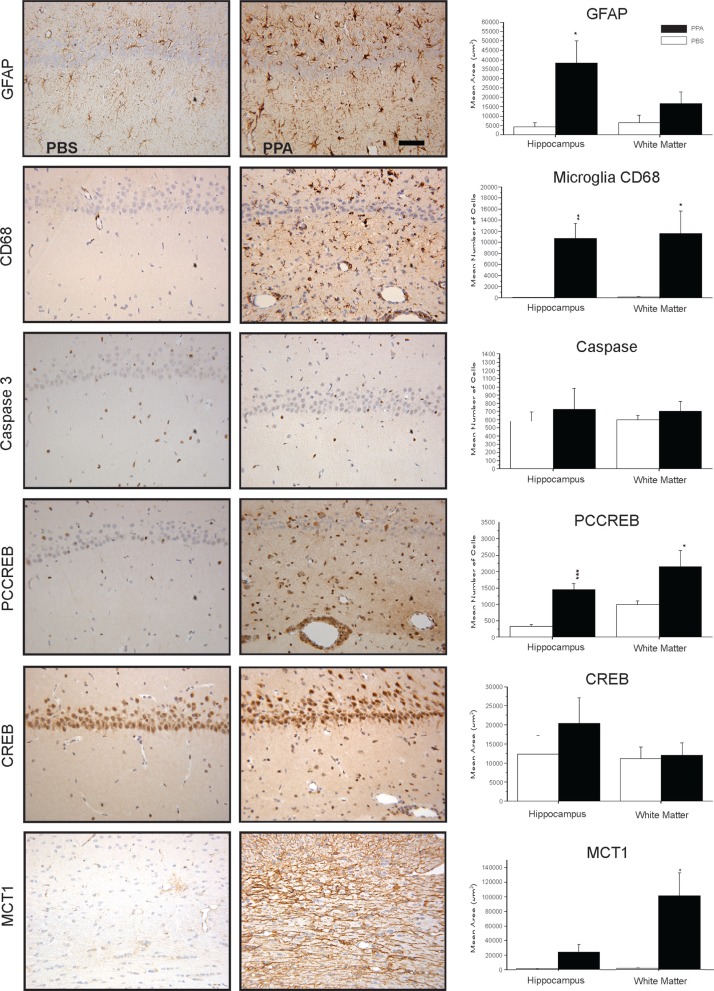
Neuropathology (avidin–biotin complex immunohistochemistry) and semiquantitative image densitometry of coronal brain sections of dorsal hippocampus (CA2) and external capsule of adult rats with 14 day BID ICV infusions of Propionic acid (PPA) or phosphate-buffered saline (PBS). PPA induced significant reactive astrogliosis (anti-GFAP) and microglial activation (anti-CD68), without apoptotic neuronal cell loss (anit-cleaved caspase 3) in rat hippocampus, similar to finding in autopsy brain from patients with autism. Nuclear translocation of anti-CREB and an increase of anti phosphoCREB immunoreactivity are observed in neural, glial, and endovascular epithelium by PPA treatment, suggestive of gene induction. PPA increases monocarboxylate transporter 1 immunoreactivity, primarily in white matter external capsule, suggestive of alterations in brain shot-chain fatty acid transport/metabolism. Black bars indicate PPA-treated animals; white bars indicate PBS (vehicle)-treated animals. Horizontal measurement bar = 100µ. Figures modified with permission from MacFabe et al. (2007); see publication for details of immunostaining procedures. Reproduced with permission from Elsevier Ltd.

We have performed a number of studies examining the neurohistological effects of PPA and its related fatty acids on innate neuroinflammatory processes (46–50). Immunohistochemical examinations of the brain tissue from PPA-treated rats (1–14 days), demonstrated by DAB immunostaining and semiquantitative image densitometry, show a neuroinflammatory response, characterized by increased activated microglia and reactive astrogliosis in some brain areas, including the hippocampus, cingulate, neocortex, and white matter. Interestingly these effects occur in the absence of apoptotic neuronal loss, as measured by the staining of cleaved caspase 3 (Fig. 5).

These findings are consistent with those from autopsy cases from patients with ASDs, regardless of age of death, indicating that an ongoing inflammatory process may be present throughout the life of the individual (13, 164). Microglia are a heterogeneous family of cells of myeloid lineage, which migrate from the bone marrow to the CNS both pre- and postnatally, and play a currently underappreciated role in neurodevelopmental disorders (165). When activated, they produce high levels of proinflammatory cytokines and reactive oxygen species such as hydrogen peroxide and nitric oxide. Interestingly, microglia also may be involved in neuroplastic responses, such as synaptic reorganization, and cortical pruning (166). Such effects are conceivable in both ASDs and the PPA model, as specific SCFA receptors exist on immune cells (88), and increased levels of cytokines such as tumor necrosis factor and macrophage chemoattractant protein are found in ASDs (13).

In addition, immunohistochemical analysis of PPA-treated brain sections showed evidence of increased immunoreactivity of the activated, phosphorylated form of cyclic-AMP responsive element binding protein (pCREB). Likewise, the results of *in vitro* studies show that SCFAs can induce pCREB in PC12 cells, leading to increased catecholamine synthesis (46, 133). CREB is interesting as this important neuroregulatory protein known to play a key role in the epigenetic expression of a number of genes implicated in neuroplasticity, addiction, movement and mood disorders, and memory acquisition (167). Thus, it is possible that increased activation of CREB-dependent epigenetic modulation of memory- or movement-related pathways following PPA administration could result in normal memory acquisition with perseverative behavior, repetitive behavior, or seizure similar to those observed in the PPA model and ASDs (46). Further to these findings, the fact that some other SCFAs and valproate, a compound with some structural similarity to PPA and a risk factor for autism, also are capable of further gene regulation via their histone deacetylase inhibition activity (90, 133, 168), makes the epigenetic effects of these compounds on ASD-related genes a subject of further study in our laboratory (169).

Preliminary results following immunostaining of brain sections with anti-monocarboxylate transporter 1 antibody (1:1000 dilution, Chemicon AB1286), a major transporter of PPA, related SCFA and ketones (79, 80, 82, 83), reveals significant increases in external capsule white matter immunoreactivity following repeated PPA infusions, suggestive of altered brain fatty acid transport. It is important to note that these neuropathological changes, although consistent with findings in ASD autopsy cases, occur hours or days following PPA administration, which is considerably later than the transient behavioral effects, which are induced within minutes and last approximate 20–30 min. Nonetheless, they may be important in the neuroplastic kindling response and perseverative learning behavior from repeated exposures of PPA and merit further study.

## Effects of PPA on lipid metabolism and mitochondrial function

Several reports have indicated that abnormal lipid metabolism may occur in ASDs and ASD-related disorders (170–175), including fatty acids, phospholipases A2, and membrane phospholipids. Clinical studies suggest improvements in core symptoms of patients with ASDs following supplementation with polyunsaturated fatty acids (PUFAs) (173, 176, 177) or cholesterol (178). PUFAs, the major lipid constituents of neuronal membranes, are absolutely essential for normal brain development and function, modulating membrane fluidity, gene expression, cell signaling, neuronal excitability, and oxidant protection, and provide a source of energy for the cells, particularly during early neurodevelopment (110, 179). Lipid composition in cellular membranes is particularly important in the formation of lipid rafts, which alter membrane fluidity and the function of membrane-bound proteins, including cell receptors and gap and tight junctions (51, 53, 107, 180).

Carnitine is a quaternary ammonium compound synthesized from the amino acids lysine and methionine principally by liver and kidney, and it is also obtained from diet. It is critical for the transport of fatty acids into the inner mitochondrial membrane for β-oxidation and energy production (181). Clinical studies have reported a relative carnitine deficiency and abnormal acylcarnitine profile (elevated short and long chain) in erythrocytes of ASDs of uncertain etiology (142, 182, 183) and a common X-linked genetic defect in carnitine synthesis in a subset of patients with ASDs (25), suggesting carnitine supplementation as a possible treatment for the disorder (20).

We thus used our PPA rodent model to examine whether there is any evidence for alterations in brain phospholipids and acylcarnitines following intraventricular infusions with PPA or butyrate (4 µl of 0.26 M solution BID×7 days) to adult rats (51, 53).

**Fig. 6:**
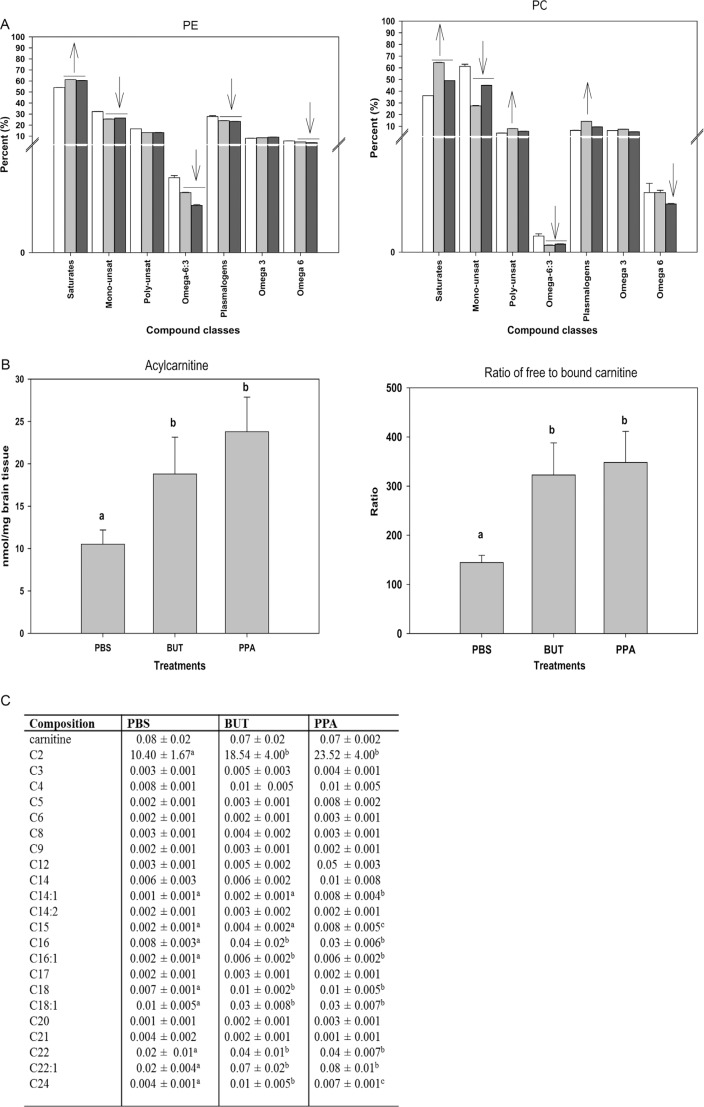
(A) Changes in phosphatidylethanolamine and phosphatidylcholine in rat brain tissue following repeated 4 µl intracerebroventricular infusions with phosphate-buffered buffer (PBS), butyric acid (BUT), and propionic acid (PPA) 0.26 M twice daily for 7 days are consistent with findings in patients with autism. Values represent means±SE. Arrows indicate significant difference between treatments [PPA and BUT compared with the control (PBS)], and direction of the changes (increase or decrease) at LSD = 0.05, *N*=6 per treatment. Monounsat = monosaturates; polyunsat = polyunsaturates. (B) Changes in acylcarnitine and ratio of bound to free carnitine in rat brain tissue following intracerebroventricular infusion with PBS, BUT, and PPA are consistent with findings in patients with autism. Values represent means±SE. Means accompanied by different superscript letters (e.g. a, b, and c) indicate significant difference between treatments at LSD = 0.05, *N*=6 per treatment. Long-chain acylcarnitine = C12–C24, short-chain acylcarnitine = C2–C9. (C) PPA and, to a lesser extent, BUT infusions increase short- and long-chain acylcarnitines, but not medium-chain acylcarnitines, similar to findings in patients with autism. Values (percent by weight) represent means±SE. Means in the same row accompanied by different superscript letters (e.g. a, b, and c) are significantly different between treatments at LSD = 0.05, *N*=9. Numbers preceding C represents carbon number of the fatty acid moiety attach to carnitine. Long-chain acylcarnitine = C12–C24, short-chain acylcarnitine = C2–C9. Figures modified with permission from Thomas et al. (2010). Reproduced with permission from Wiley Blackwell.

Brain lipid analysis was performed via GC mass spectroscopy/electrospray ionization mass spectrometry (see Thomas et al. (51, 53) for technical details). Altered lipid profiles were observed in rat brain phospholipids following infusion with both PPA and, to a lesser extent, butyrate (Fig. 6).

PPA infusion resulted in decreased levels of total monounsaturates and total ω6 fatty acids and elevated levels of total saturates in all of the studied phospholipids. In addition, a decline in total plasmalogen phosphatidylethanolamine and the ratio of ω6:ω3 was also present. Elevated levels of saturated fatty acids have been reported in red blood cells (170, 176) and plasma (184) of several autistic patients. These findings were accompanied by a concomitant decrease in total monounsaturates, particularly, 18:1n9 fatty acid. A decline in the level of this fatty acid along with several other monounsaturates (20:1n9, 22:1n9, 16:1n7, and PE 24:1n9) has also been observed in bloods drawn from patients with autism (174, 176, 185).

Carnitine and acylcarnitine analysis showed a non-significant trend toward lower free carnitine in butyrate- and PPA-treated animals; however, there was a consistent significant (*p*=0.02) increase in total acylcarnitines, total long-chain (C12 to C24) acylcarnitines, total short-chain (C2 to C9) acylcarnitines, and the ratio of free to bound carnitine following infusions with PPA and butyrate. Increases in the accumulation of short- and long-chain acylcarnitines, but not medium-chain acylcarnitines, were observed. Specifically, when these acylcarnitines were grouped according to chain length, acetyl carnitine (C2) was the major contributor to the increases observed in short-chain acylcarnitines, while C16:0, C18:0, C18:1, C22:0, and C22:1 were the major contributors to the increases observed in long-chain acylcarnitines (51, 53).

These unique acylcarnitine profiles are similar to those obtained from blood samples of patients with ASDs (182, 183) which observed elevations in the levels of some long-chain acylcarnitine species (14:1 and 14:2). Elevations in 14:1 and other long-chain acylcarnitines (16:0, 18:0, 18:1, 22:0, and 22:1) consistent with findings in our model. PPA is thought to affect mitochondrial fatty acid metabolism by binding to propionyl CoA and by sequestering carnitine (86, 186, 187). Elevation in the levels of these acylcarnitines indicates a potential metabolic disturbance by PPA infusions to affect mitochondrial metabolism in manners consistent with a unique acylcarnitine profile and a proposed environmental induction of mitochondrial dysfunction and increased oxidative stress in ASDs (51, 188).

Of particular interest, PPA is a known inhibitor of mitochondrial function, through sequestration of carnitine and the production of propionyl CoA, a potential cytotoxin (189, 190). PPA also inhibits the incorporation of acetate during lipid synthesis, an effect which is more pronounced in males (191, 192). This is interesting in light of low cholesterol levels being central to many forms of ASDs (178). Since PPA is metabolized through the mitochondrial metabolic pathways and affects mitochondrial cardiolipin levels and membrane stability (51, 53), we propose that excess exogenous PPA or its related SCFAs overwhelm mitochondrial metabolism, thereby causing mitochondrial dysfunction. Such mitochondrial dysfunction is consistent with an increase in oxidative stress. Evidence for oxidative stress has been demonstrated in the rodent PPA model of ASDs by the increase in nitric oxide-/hydrogen peroxide-producing activated microglia, coupled with the elevation of oxidized proteins and lipids, reduction of glutathione, and alteration of phospholipid/acylcarnitine profiles in brain homogenates, particularly in brain regions affected in ASDs (46, 51, 53). Such findings are all consistent with those obtained from patients with ASDs (110, 151, 152, 193, 194).

## Impairment of carnitine metabolism from a variety of causes may be central to ASD pathogenesis and regression

Although the basis of the reduced blood carnitine levels in ASDs remains unclear, we have made the observation that there are diverse clinical conditions circumstantially linked to ASDs and gastrointestinal dysfunction that show disruptions in carnitine metabolism as a common observation. There has been a growing interest in the role of carnitine in brain physiology and disease (181), particularly in brain GABAergic and astrocyte metabolism (195). As carnitine is endogenously produced from lysine and methionine, persons with defects in methylation pathways, common in individuals with ASDs (196), would have impairments in endogenous carnitine synthesis. A particularly vulnerable subgroup would be the recently discovered X-linked defect in the TMLHE enzyme, responsible for the first step in carnitine biosynthesis, which is a risk factor for non-dysmorphic autism in males (25). Collectively, these persons would rely more on dietary sources of carnitine, critical during periods of rapid development, and thus may be more sensitive to factors that impair carnitine uptake from the gut. It is known that carnitine is transported across the gut–blood and blood–brain barriers via the Na^+^-dependent organic cation/carnitine transporter 2 (OCNT_2_) (197). Carnitine transport deficits have been implicated in colitis (198) and also may impair blood–brain barrier integrity, allowing non-neurotropic influenza A virus to enter the CNS and inducing a neonatal encephalopathy (199). Interestingly, long-term administration of common antibiotics (i.e. β-lactams) for routine pediatric infections, in addition to altering gut flora favoring PPA-producing species, has been shown to directly inhibit the OCNT_2_ transporter, affecting carnitine transport across gut–blood and blood–brain barriers (197), and, thus, may contribute to a relative systemic carnitine deficiency. Additionally, antibiotics given as forms of pivalyl esters will cause an increased urinary loss of carnitine. Such interferences could be significant considering the reported high incidence of antecedent long-term antibiotic use in some patients with ASDs (65, 66, 200, 201) and the finding of unique enteric PPA-producing bacteria and gut carbohydrate malabsorbtion in regressive ASDs (65, 66, 105). This offers a potential explanation for autistic regression and also temporary behavioral improvements in some patients following vancomycin or metronidazole treatment, which transiently eradicates these bacteria (65, 66, 109, 201). However, given that Finegold has proposed eradication of ASD-causing bacteria with oritavancin and aztreonam, antibiotics which could further depress carnitine absorption, as a possible treatment of ASDs, warrants the necessity of following patient carnitine levels and providing possible carnitine supplementation in such a study (64). Furthermore, removal of refined carbohydrates from the diet, which has been suggested as an empiric treatment to improve the behavioral fluctuations and gastrointestinal symptoms in ASDs, may act by reducing substrate for these bacteria to produce PPA (46). Feeding of a high-carbohydrate diet in rats is known to increase SCFA levels and produce anxiety and aggressive behavior (139). Interestingly, preeclamptic mothers, who have an increased risk of having offspring affected by ASDs (202), have similar short- and long-chain acylcarnitine profiles (203) as found in the patients with ASDs and the PPA rodent model. Although the overall relationships remain unproven, it appears that taken collectively, we have proposed the above observations link the decreased carnitine levels in some patients with ASDs with several genetic and environmental factors consistent with regression, gastrointestinal symptomatology, microbiology, and lipid biomarkers and with experimental findings obtained using the PPA model. Furthermore, oral carnitine and its derivative acetyl-l-carnitine have both neuroprotective (181, 204, 205) and coloprotective properties (206) and deserve further investigation as therapeutic agents in developmental disorders, including ASDs (22, 46, 51, 207). These findings would also warrant more rigorous and possibly repeated carnitine/acylcarnitine screening of ‘patients at risk’, such as infants with clinical evidence of developmental delay and gastrointestinal dysfunction, particularly in the presence of maternal/infant hospital-acquired infection or long-term antibiotic use.

## Conclusions/limitations/future directions

It is important to note an animal model is unlikely to completely replicate a human disease. The usefulness of models relates to the various types of validity that can be shown to exist for specific conditions. Face validity, or the degree to which the model is able to capture the phenomenology of the disorder, is usually an essential first step in developing a model (208, 209), but additional evidence for construct validity, that is, a probable theoretical rationale for the model, possibly based on etiology is equally as important. Crawley (120) has provided a list of symptoms of ASDs that would aid in the establishment of face validity for a rodent model, along with suggested behavioral tests for such symptoms.

In the initial development of our model of ASDs, we focused on four broad aspects, such as behavioral, brain electrographic and neuropathological characteristics and biochemical markers, which would provide some face, as well as, construct validity for the model. At a behavioral level, we were interested in determining whether PPA would induce hyperactivity, stereotypies and repetitive behaviors, object preference, perseveration, and social impairment consistent with ASDs. Electroencephalographic recordings allowed us to monitor for cortical and subcortical epileptiform activity and development of seizures along with behavioral assessment of seizure and movement disorder. Finally, we also looked for possible neuropathological effects that might be consistent with those observed in humans, such as innate neuroinflammatory, astroglial and microglial changes, and neuroplastic (i.e. CREB activation, monocarboxylate transport) changes, as well as biochemical markers suggestive of increased oxidative stress, altered phospholipid/acylcarnitine profiles, and mitochondrial dysfunction.

These initial studies provide support for the face validity of the intraventricular PPA administration in adult rat model of autism, as the behavioral and electrographic changes observed resemble those seen in the human condition. The rapid induction and transient nature of these peculiar behavioral and electrographic effects, their potentiation with repeated exposures, and the absence of such behaviors in rats receiving control compounds (1-propanol) suggest the involvement of diverse PPA-activated neural mechanisms and present the first attempt in the field to model the possible fluctuating, as opposed to static behavioral course in ASDs.

Evidence from human studies suggests that ASD is a condition that represents an ongoing neuroinflammatory or neurometabolic disorder possibly resulting from an increased sensitivity to oxidative stress or acquired mitochondrial dysfunction from a variety of environmental risk factors. The neuropathological and biochemical findings of our model support this hypothesis. Interestingly, the observed impairments in the glutathione system, increased oxidative stress, and altered phospholipids/acylcarnitine profiles, suggestive of mitochondrial dysfunction in our model, are consistent with the findings in human autism cases and would provide a plausible mechanism for increased environmental sensitivity to a variety of agents. The similarities in neuropathological and biochemical changes between the animal model and human ASD cases, coupled with the presence of PPA-producing bacteria, could represent similar underlying etiological processes subsequent to central nervous system insult with PPA. However, much additional work needs to be done before these similarities can be taken as support for construct validity of the animal model.

It is important to note, other than reports of increased PPA in stool samples, studies are lacking (210) that have systematically examined PPA and its related metabolites in ASDs. The short half-life and rapid metabolism via β oxidation, incorporation into acylcarnitines and other lipids, and intracellular concentration of PPA via specific transporters and pH-dependant mechanisms also make definitive measurement and interpretation difficult. However, there is some indirect evidence in patients with ASDs suggestive of increased PPA, including a relative carnitine deficiency, unique phospholipid and short- and long-chain acylcarnitine profiles, elevations of nitric oxide metabolites, and oxidative stress markers, known to be elevated by PPA in experimental systems, including our rodent model. The underappreciated incidence of complex gene polymorphisms in propionic acidemia in some populations, a potential population of partial metabolizers of PPA, and documented ASD symptoms in some of these patients, is interesting. The antiepileptic drug valproate, a prenatal risk factor for autism, alters both fatty acid and biotin metabolism and causes the depletion of carnitine stores each of which could theoretically increase PPA levels. Prenatal exposures to ethanol, known to produce developmental delay, also increases PPA levels to millimolar levels putatively by depleting intracellular carnitine stores.

Despite the lack of studies on PPA and its related fatty acids in ASDs, abnormalities of lipid metabolism and mitochondrial dysfunction have been postulated as a potential cause of the condition. Patient studies have noted impairments in mitochondrial β-oxidation of fatty acids, reductions in essential PUFAs levels, and anecdotal reports of improvement in some patients with essential fatty acid and carnitine supplementation.

Likewise, the intriguing finding that long-term administration of common antibiotics for routine infections not only may favor PPA-producing bacteria but also may impair colonocyte function and directly inhibits gut carnitine transport, leading to an acquired mitochondrial encephalopathy, should certainly be investigated as a key factor in ASD regression. Notably, screening for unique enteric bacterial populations, plasma phospholipids/acylcarnitines, inherited carnitine transport defects, ‘partial metabolizers’ of PPA (i.e. organic acidemias, mitochondrial disorder), and gut dysfunction deserves further examination. This may be useful not only in patients with ASDs but also in ‘persons at risk’, including individuals in neonatal units, those with immune deficiencies and patients on long-term antibiotic usage. Likewise, to follow these biomarkers in response to proposed treatments (eradication of PPA-producing bacteria, probiotics, dietary carbohydrate restriction, carnitine supplementation) in animal models and ultimately patients would prove valuable. Importantly, the longitudinal examination of the infant gut microbiome, characterizing not only the presence, but also the absence, of specific gut bacterial population in infants who progress neurotypically or subsequently go on to develop ASDs will be critical. Novel bacterial species, such as *Desulfovibrio*, and their ability to produce H_2_S, a possible mitochondrial toxin, possessing both colonotoxic and neurotoxic effects synergistic to PPA, and their interaction with *Clostridia* and other members of the gut microbiome deserve further investigation. Lastly the neurobiological effects of other short chain fatty acids, their derivatives and the array of other metabolites of the microbiome remain largely unexplored.

In a broader context, it is intriguing to postulate that microbiota, through natural selection, have evolved to use their metabolites to modulate the physiology and ultimately behavior of the host, to promote survival. This has been documented in behavioral neurobiology, with such examples as cordyceps fungus producing climbing behavior in ants, and borna and rabies viruses eliciting salivary transmission and biting behavior in mammals (211, 212). It has recently been established that transplantation of fecal material in germ-free mice can alter brain gene expression and phenotypic behavior of the host (213). In light of this, the observation of restrictive eating of carbohydrates, diarrhea and fecal smearing in patients with ASDs which could theoretically promote organism growth and spread, is intriguing. It is also worth noting that many of the effects of lower doses of SCFAs on gut physiology and immune function are indeed beneficial to host and ultimately bacterial survival. Finally, it is important to note the ability of SCFAs to elicit anxiety-like, perseverative, repetitive, ritualistic, and antisocial behaviors that are common to many other neuropsychiatric conditions such as obsessive compulsive, anxiety, attention deficit/hyperactive, mood, and eating disorders, irritable bowel syndrome, pediatric autoimmune neuropsychiatric disorder associated with streptococcal infections (PANDAS), and schizophrenia, where infectious agents have been proposed (214–216).

The growing incidence of ASDs and ASD-related conditions, coupled with the observed alterations in the human microbiome secondary to dietary, medical, and agricultural factors, and their potential effect on human and animal behavior, should be further examined. The impact of human migration and urbanization, domestication of plant and animals, and resultant human diseases shaping cultures is not trivial and has been discussed elsewhere (217).

**Fig. 7:**
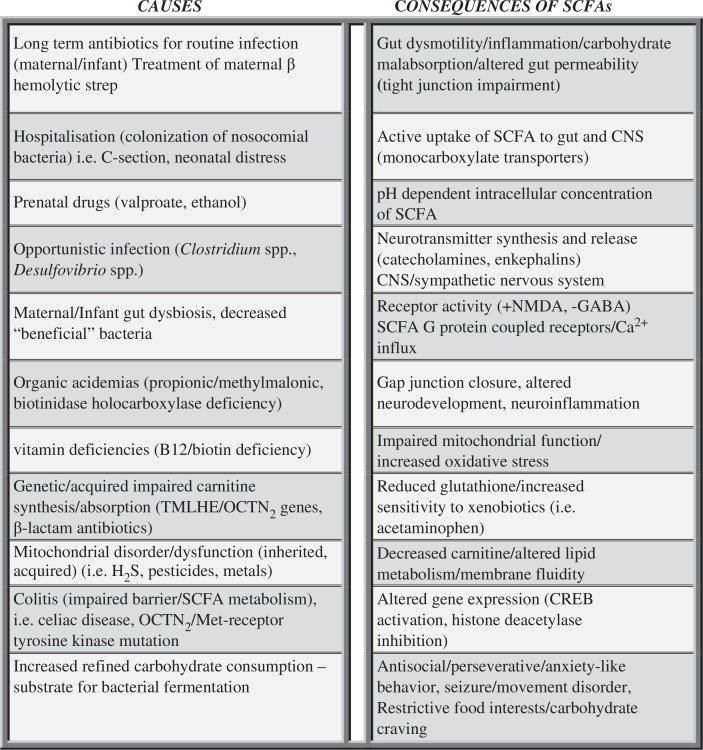
Potential causes and consequences of increased enteric short-chain fatty acid production and/or decreased breakdown and their relation to autism spectrum disorder. These findings, which are not mutually exclusive, may contribute to the pathophysiology, behavioral symptoms, and comorbidities of autism.

In conclusion, these present studies examined the effects of intracerebroventricular PPA in adult rats and their relation to the pathophysiology of ASDs. Given the intriguing multiple effects of PPA on many neurological, gastroenterological, metabolic, and immunological processes, together with our initial findings on behavior, electrophysiology, neuropathology, and biochemistry, the rat intraventricular PPA model maybe a useful paradigm for the examination of the disparate symptoms and pathophysiology of ASDs (Fig. 7).

However, notwithstanding of the remarkable effects of these compounds on an ‘intact’ adult brain, autism is, of course, a neurodevelopmental disorder, with evidence of altered nervous system development, involving white matter abnormalities and disorders of neuronal microcircuitry. Treatments of rodents with PPA, other SCFAs and appropriate control compounds, and ultimately infection of ASD-associated bacteria during critical times of pre- and postnatal development are essential steps in extending the validity of this animal model. Such studies using systemic and dietary exposure at these time periods are ongoing in our laboratory and our collaborators (52, 130, 218).

Although compelling, further research examining PPA and its related SCFAs is still needed at both the basic and the clinical levels to better understand the underlying mechanisms of how these and other metabolic products of the host microbiota may modulate host physiology throughout the lifecycle and whether they are directly involved in the disparate brain and behaviors of ASDs and ASD-related conditions. Conceptually, it is the author's opinion that the pathophysiology of ASDs may be more completely understood as being similar to conditions such as ethanol intoxication, or diabetes, and the resultant complex interactions between diet, genetics, metabolism, host microbiome, and behavior, that are well known to exist in these treatable disorders throughout the life cycle. Considering the marked increase, considerable morbidity, and social burden of ASDs, collaboration in the fields of microbiology, clinical and basic neuroscience, immunology, biochemistry, gastroenterology, obstetrics, genetics, and epidemiology is warranted and should be encouraged.
